# Dr. Theophilus Parvin

**Published:** 1898-03

**Authors:** 


Dr. Theophilus Parvin died at his residence in Philadelphia, January 29, 1898, aged 69 years. His death was attributed to cardiac asthma, complicated by uremia and edema of the lungs. It was thought, however, that he would recover even up to a very late day of his illness.

Dr. Parvin was born at Buenos Ayres, A. R., January 9, 1829. His father, who was a presbyterian missionary, sent him to this country to be educated while he was yet a mere lad, and he entered the preparatory department of Lafayette college when 11 years of age. A few years afterward he entered the Indiana State University, from which he graduated in 1847. Afterward he taught school at Lawrenceville, N. J., studied Hebrew in Princeton theological seminary, and entered the medical department of the University of Pennsylvania, from which he took his doctorate degree in 1852. He served as resident physician at Wills Eye hospital, as surgeon on the steamer Tonawanda—a New York and Liverpool packet—and then removed to Indianapolis, where he remained in practice until 1864. He was then elected professor of materia medica at the Ohio Medical College, Cincinnati; in 1869 he became professor of obstetrics in a medical school at Indianapolis and later held the same chair in the University of Louisville. In 1883 he removed to Philadelphia, having been chosen professor of obstetrics and diseases of women and children in Jefferson Medical College.

Dr. Parvin was a member of many American and foreign medical societies, of some of which he had been president. He was an accomplished teacher, a delightful conversationalist and a man of great scholarly attainments. He had a decided taste for literature and some of his contributions to the medical periodical press are marked for their pure English, elegant diction and finished rhetoric. We recall at this time, especially, his review of Goodell's first edition of Lessons in Gynecology, that appeared in the *American Journal of Medical Sciences*. Parvin's treatise on the science and art of obstetrics, a well-known text-book, is now passing through its third edition, and he was also the American editor of Winkel's diseases of women, both of which have been reviewed in the columns of the Journal. Dr. Parvin has left the impress of his talent upon the annals of American medicine, especially of obstetric medicine, quite as markedly as any man of his time.

He is survived by a daughter, Mrs. James Philip Baker, of Indianapolis, and two sons, Mr. Theophilus Wylie Parvin, of Pittsburg, and Dr. Noble Butler Parvin, who is now engaged in the practice of medicine in Philadelphia.

